# Syphilis in riverine communities: prevalence and associated factors*

**DOI:** 10.1590/1980-220X-reeusp-2021-0258

**Published:** 2022-01-07

**Authors:** Wynne Pereira Nogueira, Matheus Figueiredo Nogueira, Jordana de Almeida Nogueira, Maria Eliane Moreira Freire, Elucir Gir, Ana Cristina de Oliveira e Silva

**Affiliations:** 1 Universidade Federal da Paraíba, Programa de Pós-Graduação em Enfermagem, João Pessoa, PB, Brazil.; 2 Universidade Federal de Campina Grande, Centro de Educação e Saúde, Cuité, PB, Brazil.; 3 Universidade de São Paulo, Escola de Enfermagem de Ribeirão Preto, Ribeirão Preto, SP, Brazil.

**Keywords:** Syphilis, Vulnerable Populations, Prevalence, Risk Factors, Sexually Transmitted Diseases, Sífilis, Poblaciones Vulnerables, Prevalencia, Factores de Riesgo, Enfermedades de Transmisión Sexual, Sífilis, Populações vulneráveis, Prevalência, Fatores de Risco, Doenças Sexualmente Transmissíveis

## Abstract

**Objective:**

To estimate the prevalence of syphilis and associated factors in riverine communities.

**Method:**

This is a cross-sectional and analytical study carried out with 250 riverside dwellers living in five communities in the city of João Pessoa, state of Paraíba. Data were collected through interviews and rapid screening tests to investigate syphilis. Bivariate, logistic regression and weight of evidence analysis were performed to identify the association between risk factors and behavior variables and rapid test positivity.

**Results:**

he prevalence of syphilis was 11.6% (95%CI: 7.5–15.6). Riverside dwellers who have a previous history of Sexually Transmitted Infection (OR 8.00; 95%CI: 2.76–23.2), history of imprisonment (OR 7.39; 95%CI: 1.61–33.7) and who reported having more than two sexual partners in the last 12 months (OR 4.31; 95%CI: 1.55–11.9) were more likely to be positive for syphilis.

**Conclusion:**

High prevalence of syphilis among riverside dwellers and the presence of behavioral factors that increase vulnerability to acquiring the infection. The need to invest in preventive and screening strategies for syphilis in populations considered vulnerable is highlighted.

## RESUMO

**Objetivo:**

estimar a prevalência de sífilis e fatores associados em comunidades ribeirinhas.

**Método:**

estudo transversal e analítico realizado com 250 ribeirinhos residentes em cinco comunidades no município de João Pessoa, estado da Paraíba. Os dados foram coletados por meio de entrevista e testes rápidos de triagem para investigação da sífilis. Realizaram-se análise bivariada, de regressão logística e análise do peso da evidência (*Weight of Evidence*) para identificar a associação entre as variáveis de fatores e de comportamento de risco e a positividade do teste rápido.

**Resultados:**

a prevalência de sífilis foi de 11,6% (IC95%: 7,5–15,6). Ribeirinhos que possuem história prévia de Infecção Sexualmente Transmissível (OR 8,00; IC95%: 2,76–23,2), histórico de prisão (OR 7,39; IC95%: 1,61–33,7) e que relataram ter mais de dois parceiros sexuais nos últimos 12 meses (OR 4,31; IC95%: 1,55–11,9) apresentaram maiores chances de apresentar resultado positivo para sífilis.

**Conclusão:**

alta prevalência de sífilis entre os ribeirinhos e presença de fatores comportamentais que aumentam a vulnerabilidade à aquisição da infecção. Reforça-se a necessidade do investimento de estratégias preventivas e de rastreamento de sífilis em populações consideradas vulnerabilizadas.

## RESUMEN

**Objetivo:**

estimar la prevalencia de sífilis y factores asociados en comunidades ribereñas.

**Método:**

estudio transversal y analítico realizado con 250 ribereños residentes en cinco comunidades en el municipio de João Pessoa, Paraíba, Brasil. Los datos fueron recolectados por medio de entrevista y pruebas serológicas rápidas de triaje para investigación de sífilis. Se realizaron análisis bivariado, análisis de regresión logística y análisis del peso de la evidencia (Weight of Evidence) para identificar la asociación entre las variables de factores y de comportamiento de riesgo y la positividad de la prueba serológica rápida.

**Resultados:**

la prevalencia de sífilis fue un 11,6% (IC95%: 7,5–15,6). Ribereños que poseen historia anterior de Infección Sexualmente Transmisible (OR 8,00; IC95%: 2,76–23,2), histórico de cárcel (OR 7,39; IC95%: 1,61–33,7) y que relataron tener más de dos parejas sexuales en los últimos 12 meses (OR 4,31; IC95%: 1,55–11,9) demostraron mayores riesgos de presentar resultado positivo para la enfermedad.

**Conclusión:**

alta prevalencia de sífilis entre los ribereños y presencia de factores comportamentales que aumentan la vulnerabilidad a la adquisición de la infección. Se refuerza la necesidad de inversión en estrategias preventivas y de identificación de sífilis en poblaciones consideradas vulnerables.

## INTRODUCTION

Syphilis remains a serious public health problem and is still a challenge to health systems around the world. It is characterized as an infection of chronic progression, caused by the bacteria *Treponema pallidum*, transmitted mainly through sex (oral, vaginal, and anal), blood, and vertically. It is sometimes asymptomatic, which makes the transmission chain control a challenge – especially if inadequately treated^(1)^.

The World Health Organization (WHO)^(2)^ estimated the occurrence of 6.3 million new cases of syphilis in the world. In Brazil, even though syphilis is curable and there is low-cost treatment available in the public network, data show extremely high growth curves for the disease since it has become a mandatory notification infection.

In the country, in 2019, the detection rate of acquired syphilis was 72.8 cases per 100,000 inhabitants, with notification of 152,915 cases^(3)^. In the state of Paraíba, there was an increase from 8.2 cases in 2015 to 49.1 in 2019, with the capital João Pessoa showing a higher detection rate than the national one: 124.0 cases of acquired syphilis per 100,000 population^(4)^.

Considering these data, the increase in the occurrence of syphilis may be related to the growth in the number of notifications and improvement of the epidemiological surveillance system^(5)^, as well as factors related to social, economic, biological, cultural aspects and, above all, to the population behavioral changes^(5,6)^.

Furthermore, the prevalence of syphilis worldwide in key populations is high, especially in men who have sex with men (MSM), sex workers, and injecting drug users. However, the incidence of infection also has a dissemination character in populations considered vulnerable, which represent communities with social, economic, and cultural weaknesses that increase the risk of contamination of Sexually Transmitted Infections (STIs)^(7,8)^.

Low adherence to condom use, multiple partners, and injecting drug use may represent individual vulnerabilities related to risky sexual behaviors that increase the incidence of syphilis^(7,9)^. In addition, the influence of socioeconomic aspects, such as low education, low income, and difficult access to health services, are also determining factors for maintenance and/or the emergence of an infection^(10)^.

In this context, the riverside dwellers are included, as they have situations of vulnerability that make them weak regarding issues related to morbidity and health care, especially sexual health^(11)^. Riverine populations in the urban area of northeastern Brazil are those who live on the riversides of the main rivers crossing the cities, in a space of subnormal agglomerations, difficult access, with inadequate living conditions and lack of basic sanitation sewage systems^(12)^.

Thus, they are individuals exposed to determinants and conditions related to the health-disease process, such as low socioeconomic conditions, limited access to health services, as well as limitations related to geographic and organizational factors^(13)^.

In Brazil, data on the prevalence of STIs in riverside populations are scarce. For these people, issues related to waterborne diseases and injuries predominate when it comes to knowledge production. Therefore, knowing the prevalence of syphilis and the factors associated with its acquisition can contribute to the planning of health strategies and actions that minimize the potential factors and risk behaviors determining the infection in this population group, as well as contributing to the expansion of knowledge about the epidemiological profile of this population.

Given the above, this study aimed to estimate the prevalence of syphilis and associated factors in riverine communities in the state of Paraíba.

## METHOD

### Design of Study

This is a cross-sectional and analytical study.

### Local

The study was carried out in five riverside communities – São José, Tito Silva, Porto do Capim, São Rafael and Comunidade do “S” – located in the municipality of João Pessoa and in surrounding areas in the state of Paraíba.

### Population

The target population consisted of residents of the aforementioned riverside communities. Individuals aged 18 years or over were included, and those who had more than one residence, where at least one of them was not located in the investigated community, were excluded.

For the composition of the sample, 11,498 individuals were considered, which corresponds to the sum of the total number of residents of these communities: São Rafael (n = 1,800), São José (n = 7,078), Tito Silva (n = 1,140), Porto do Capim (n = 550), and Comunidade do S (n = 930). To determine the sample size, a confidence interval of 95%, a desirable margin of error of 5.4%, and an estimated frequency of 26.15% were assumed^(14,15)^. Thus, the study sample consisted of 250 individuals.

### Data Collection

Data collection was carried out from June to October 2019. The articulation for the operationalization of the study was made through the community’s family health teams with the support of the Community Health Agents (*ACS*). Eligible individuals were invited to participate in the research through prior contact carried out by the ACSs. Those who accepted were informed about the importance of the study, the objectives, risks and benefits related to their participation. Soon after, an individual and private interview was carried out at support sites within the communities.

Information was collected from a structured questionnaire, adapted from the instrument used in the Survey of Knowledge, Attitudes and Practices in the Brazilian Population^(16)^, covering sociodemographic data and possible behavioral factors for the acquisition of syphilis, as well as the use of the Alcohol Use Disorders Identification Test (AUDIT). AUDIT is an instrument developed by WHO^(17)^ and used to measure the pattern of alcohol use in the last 12 months by an individual. The riverside dwellers who obtained scores from 0 to 7 on the AUDIT were considered low-risk consumers and those who obtained results equal to or above 8 were classified in a pattern of harmful and problematic alcohol consumption (risk, harmful, and of probable dependence consumption).

After the interview, the participants were invited to perform the rapid screening test for syphilis, which in this study was the lateral flow immunochromatographic assay (Bioclin: Quibasa Química Basica, Brazil), responsible for the qualitative detection of anti-*Treponema pallidum* total antibodies, with the serological collection obtained through digital pulp blood sampling. For its performance, all the guidelines from the manufacturer and the Ministry of Health protocol were strictly followed^(18)^, including pre-test and post-test counseling, individually and privately, regardless of a positive or negative result for syphilis.

Participants with reactive results were guided and referred to the city’s STIs reference service for monitoring and free individual treatment. The team of interviewers responsible for the rapid testing and the interview consisted of graduate and undergraduate students and health professionals (nurses), who were previously trained and qualified by technicians from the State Health Department, as directed by the Ministry of Health^(18)^.

### Data Analysis and Treatment

The collected data were double-entered into a Microsoft Excel 2010 spreadsheet and imported into the statistics software SPSS version 20 to perform descriptive and inferential analyses. The prevalence of syphilis, investigated according to positivity for the rapid test, was calculated considering a 95% confidence interval (95%CI). The positivity of the rapid test for syphilis was considered as a dependent variable.

To investigate the association between sociodemographic and behavioral variables and the positivity of the rapid test for syphilis, a bivariate analysis was performed, carried out using Pearson’s chi-square test and Fisher’s exact test. The variables that presented a significance level of p < 0.20 were simultaneously included in the binary logistic regression model, generating the odds ratio with 95% CI. In the final model, the variables showing a statistically significant association with p ≤ 0.05 were considered.

After applying the logistic regression model, the Weight of Evidence analysis was performed to determine the strength of the relationship between the independent and dependent variables, considering that, in an Information Value (IV) <0.02, the predictor is too weak (not useful); from 0.02 to <0.1, the predictor has a weak relationship; from 0.1 to 0.3, an average strength ratio; and >0.3, the predictor has a strong relationship^(19)^.

### Ethical Aspects

All ethical precepts that guide the research involving human beings, established in Resolution No. 466/2012 of the National Health Council, were followed, and all participants signed the Free Informed Consent Term (FICT). The research was approved by the Research Ethics Committee of the Universidade Federal da Paraíba with opinion no. 3.340.273/2019.

## RESULTS

Of the 250 riverside dwellers interviewed in the study, there was a predominance of females, 170 (68.0%); from the age group between 18 and 39 years, 108 (43.2%); and with up to eight years of schooling, 155 (62.0%). As for marital status, most participants declared themselves married or in cohabitation, 160 (64.0%), and with a monthly family income of up to 1.5 minimum wages, 208 (83.2%). Regarding the riverside communities, 154 (61.6%) individuals live in the community São José, 39 (15.6%) in São Rafael, 25 (10.0%) in Tito Silva, 20 (8.0%) in Comunidade do S, and 12 (4.8%) individuals in Porto do Capim.

The prevalence of syphilis, investigated according to positivity of the rapid test, was 11.6% (95%CI: 7.5–15.6), which corresponds to 29 riverside dwellers who tested positive for this infection.

Table 1 shows the bivariate analyses of sociodemographic characteristics and their association with rapid syphilis test positivity.

**Table 1.** Association between sociodemographic characteristics and the positivity of the rapid test for syphilis performed in residents of riverside communities – João Pessoa, PB, Brazil, 2019.

Variables	Rapid syphilis test		p-value*
	Positive (n = 29) n (%)	Negative (n = 221) n (%)	
**Sex**			
Male	11 (13.8)	69 (86.2)	0.460
Female	18 (10.6)	152 (89.4)	
**Age**			
18 to 39 years old	12 (11.1)	96 (88.9)	0.601
40 to 59 years old	10 (10.2)	88 (89.8)	
≥60 years old	7 (15.9)	37 (84.1)	
**Level of education**			
≤8 years of study	19 (12.3)	136 (87.7)	0.670
>8 years of study	10 (10.5)	85 (89.5)	
**Marital status**			
Married/Consensual Union	14 (8.8)	146 (91.2)	0.262
Single/Separated/Widowed	15 (16.7)	75 (83.3)	
**Monthly income**			
≤1 minimum wage	18 (10.5)	154 (89.5)	0.401
>1 minimum wage	11 (14.1)	67 (85.9)	

* Chi-Square Test.

In the association between the variables of the main risk behaviors associated with the positivity of the rapid test for syphilis, the number of sexual partners (p = 0.012), the previous history of STI (p < 0.001), smoking (p = 0.020), AUDIT score (p = 0.023), and history of imprisonment (p = 0.026) were statistically significant (Table 2).

**Table 2.** Association between the main risk behaviors with the positivity of the rapid test for syphilis performed in residents of riverside communities in João Pessoa, PB, Brazil, 2019.

Variables	Rapid syphilis test		p-value*
	Positive (n = 29) n (%)	Negative (n = 221) n (%)	
**Age of first sexual intercourse**			0.073**
≤15 years	18 (16.1)	94 (83.9)	
>15 years	11 (8.0)	127 (92.0)	
**Number of sexual partners in the last 12 months**			0.012**
0 to 1 partner	16 (8.5)	172 (91.5)	
2 or more partners	13 (21.0)	49 (79.0)	
**Sexual intercourse with a person of the same sex**			0.079^†^
Yes	5 (23.8)	16 (76.2)	
No	24 (10.5)	205 (89.5)	
**Knows male condom**			0.265^†^
Yes	25 (10.9)	205 (89.1)	
No	4 (20.0)	16 (80.0)	
**Knows the female condom**			1.000**
Yes	19 (11.4)	147 (88.6)	
No	10 (11.9)	74 (88.1)	
Condom use in the last sexual intercourse*			0.402**
Yes	6 (15.4)	33 (84.6)	
No	18 (10.4)	155 (89.6)	
**Sexual intercourse with a sex worker**			0.777^†^
Yes	5 (14.3)	30 (85.7)	
No	24 (11.2)	191 (88.8)	
**Received money or paid in exchange for sex**			0.051**
Yes	8 (21.6)	29 (78.4)	
No	21 (9.9)	192 (90.1)	
**Sexual intercourse with a partner met through cell phone call**			1.000^†^
Yes	2 (15.4)	11 (84.6)	
No	27 (11.4)	210 (88.6)	
**Previous history of STIs**			<0.001**
Yes	14 (26.9)	38 (73.1)	
No	15 (7.6)	183 (92.4)	
**Smoking**			0.020**
Yes	13 (19.4)	54 (80.6)	
No	16 (8.7)	167 (91.3)	
**Illegal drug use**			0.071**
Yes	11 (18.0)	50 (82.0)	
No	18 (9.5)	171 (90.5)	
**AUDIT score**			0.023**
<8 points	22 (9.9)	201 (90.1)	
≥8 points	7 (25.9)	20 (74.1)	
**History of imprisonment**			**0.026^†^**
Yes	5 (31.2)	11 (68.8)	
No	24 (10.3)	210 (89.7)	

* Those who had sexual intercourse in the last 12 months; ** Chi-square test; ^†^ Fisher’s exact test.

Logistic regression analysis showed that riverside dwellers with a previous history of STIs (OR 8.00; 95%CI: 2.76–23.2), history of imprisonment (OR 7.39; 95%CI: 1.61–33.7), and with a report of more than two sexual partners in the last 12 months (OR 4.31; 95%CI: 1.55–11.9) are approximately eight, seven and four times more likely to have a positive rapid test result for syphilis, respectively (Table 3).

**Table 3.** Odds ratios for the significant variables identified by logistic regression for a positive rapid test result for syphilis in riverside communities in João Pessoa, PB, Brazil, 2019.

Variables	Odds ratio	95%CI	p-value*
Previous history of STIs	8.00	2.76–23.2	<0.001*
History of imprisonment	7.39	1.61–33.7	0.010*
More than two sexual partners in the last 12 months	4.31	1.55–11.9	0.005*

95%CI: 95% confidence interval; * Significant result with p < 0.05.

**Figure 1.**
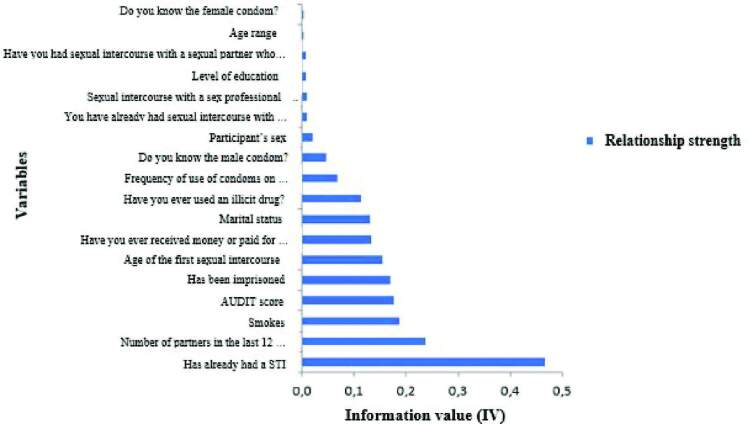
Result of the outcome analysis via classification by Information Value (IV) generated by the *WoE*. João Pessoa, PB, Brazil, 2019.

After applying the logistic regression model, the Weight of Evidence analysis was performed, as shown in Figure 1.

The WoE analysis showed that the same variables detected by the logistic regression model also showed evidence of being associated with the outcome: multiple partners (IV = 0.237), history of imprisonment (IV = 0.169) and, in particular, the variable related to the fact of having a previous history of STIs (IV = 0.467).

## DISCUSSION

The increase in syphilis cases in Brazil shows a growing trend over the last ten years^(3)^. However, investigation of the infection in riverside populations is still scarce, especially in those living in the Northeast region. The few studies available on STIs in this population group were carried out in the Brazilian Amazon^(8,20,21)^. Thus, this study represents one of the first on the national scenario to estimate the prevalence of syphilis in the riverside population living in urban areas.

Due to the lack of data on the estimated prevalence of syphilis and factors associated with the infection in these individuals, the comparison with riverside realities will be limited. Thus, for comparative analyses, other populations characterized as vulnerable in health-related aspects will be considered.

As for the sociodemographic aspects of the 250 residents interviewed in the riverside communities, it was observed that most have up to eight years of education and an income of up to 1.5 minimum wages. The low education and low income found are common characteristics in riverside populations from other locations^(12,13,21)^.

In this study, through the positivity of rapid tests, it was possible to estimate a prevalence of syphilis of 11.6% (95%CI: 7.5–15.6) in the riverside population investigated. This identified rate was higher than the national estimate of syphilis^(3)^, 0.5% (95%CI: 0.4–0.6). Furthermore, in Paraíba, in 2019, 1,973 cases of acquired syphilis were reported, with an increase in the detection rate from 39.7 cases to 49.1^(4)^. In addition, it is observed that studies involving other vulnerable groups had a lower prevalence compared to the population of this research^(20,22)^.

In this perspective, a study carried out with 153 women in the Tapajós region, in the Brazilian Amazon, showed a global prevalence of syphilis of 3.3% (95% CI: 1.40–7.42)^(20)^. In the same region, a survey carried out with 45,967 indigenous people showed a prevalence of 1.82% (95%CI: 1.69–1.94)^(22)^ for this infection, which corresponds to low prevalence compared to the riverside population. Another investigation carried out with 416 illegal miners in French Guiana found a prevalence of 11.5% (95%CI: 8.5–14.6) for syphilis^(23)^, prevalence similar to the population of this research.

Thus, the result of the present research shows a high prevalence of syphilis in riverside dwellers, which is concerning. In addition to the fact that the infection has asymptomatic characteristics, encouraging silent propagation, the population has limited access to health services, which hinders the diagnosis and treatment. In addition to these conditions, the research also shows factors and behaviors among riverside dwellers that may favor the spread of syphilis.

It is observed that, although the sociodemographic characteristics of riverside dwellers have not been statistically significantly associated with the prevalence of syphilis, it is believed that education and economic status are determining factors in the health-disease process. Individuals having low education and low economic conditions reflect in less access to information about health care, risk perception and prevention of multiple diseases^(10,24)^. Therefore, they are individuals considered to be more susceptible to acquiring STI.

Furthermore, the analysis of risky sexual behaviors showed that the multiplicity of partners increases by 4.3 (95%CI: 1.55–11.9) the chance that riverside dwellers present a positive rapid syphilis test. It is known that the multiplicity of partners is a risk behavior widely known for the risk of acquiring an STI^(25)^.

Similar data was found in a study carried out in municipalities in the state of Goiás, Brazil, with 481 homeless men, in which the multiplicity of partners was statistically associated with a positive result for syphilis^(26)^. Such behavior may be related to the early beginning of sexual life and the influence of socioeconomic issues^(10,27)^. In addition, the lack of knowledge and low adherence to condom use can favor the acquisition and spread of the infection^(27)^.

This fact is seen in the study population, as the results show that 69.2% of riverside dwellers reported not having used a condom in the last sexual intercourse. This may be a consequence of the low educational level of individuals residing in these communities, since educational issues, including the level of understanding and self-care capacity, can influence adherence to barrier methods and less concern with health care^(27)^.

In this investigation, 6.4% of the riverside dwellers reported a history of imprisonment. It is essential to address this data, since contexts directly linked to social vulnerability – such as poverty, unemployment and socioeconomic exclusion – can promote the increase in crime in the country^(28)^, which can be observed in riverside realities, thus leading to the need to implement public policies for vulnerable populations.

Moreover, this risk factor was associated with the studied outcome. Riverside dwellers who reported a history of imprisonment at some point in their lives were approximately seven times more likely (OR 7.39; 95%CI: 1.61–33.7) to have a positive rapid test result for syphilis. Research identifies that prisons provide a favorable epidemiological scenario for increasing STI rates due to the prison population’s high-risk behavior, such as sexual intercourse without condom use, sexual violence, in addition to sharing of sharp objects, especially for the use of injecting drugs^(9,29)^.

In the present study, the previous history of STIs was strongly associated with the outcome, according to the WoE. The results also showed that riverine populations who reported a previous history of STIs are eight times more likely (OR 8.00; 95%CI: 2.76–23.2) to have a positive result of the rapid test for syphilis. A similar association was also found in studies with other vulnerable populations^(7,30)^.

A population-based survey conducted in rural China, with 2,044,126 women, showed an association between a previous history of STIs and a positive result for syphilis (OR 27.17, 95%CI: 20.44–36.11)^(30)^. A study carried out with 1,405 homeless people in the state of São Paulo also showed this association (OR 2.6; 95%CI 1.7–4.0)^(7)^.

Late diagnosis, inadequate treatment, nonuse of condoms, multiple partners, the presence of wounds in the genital region, untreated sexual partners, reinfection and possible drug resistance are factors that may be related to the previous presence of STI in this population and, consequently, a greater vulnerability to the acquisition of syphilis^(25–27)^. It is also important to highlight that the presence of STIs increases by 18 times the risk of a person being infected with HIV, an infection for which there is no cure, so that health authorities focus greater efforts on screening, prevention, and control of the virus.

This strong association shows that making a timely diagnosis and proper treatment are essential. Biomedical, behavioral, and structural interventions centered in the specificity of vulnerable populations are essential for an effective response to the syphilis and other STIs epidemic.

An important fact refers to the high prevalence of syphilis in riverside dwellers of dry land (urban area), which is higher than the state and even national prevalence. This result reinforces the presence of social and health vulnerabilities that favor the spread of STIs among riverside dwellers and that actions aimed at the specificities of population groups in the context of STIs have to be effective.

As for the limitations of this research, the design of the cross-sectional study does not allow making causal inferences; however, it allows exploring associations and raising hypotheses. Another limitation was the estimate of syphilis prevalence investigated through the rapid test, since it does not provide information of diagnosis of active syphilis or serological scarring in previously treated people. Moreover, the situational diagnosis of the population is the first step to propose interventions and the rapid test represents a rapid diagnostic tool to be used in specific situations, such as in places with difficult access to health services and in vulnerable population segments, besides providing information to health managers for possible interventions related to preventive measures and the epidemiological profile of morbidity in a given population.

## CONCLUSION

The research provides evidence of the high prevalence of syphilis, according to rapid test positivity, in riverine communities, and that a previous history of STI, history of imprisonment, and multiple partners were behavioral factors associated with a greater chance of riverine populations to have a positive rapid syphilis test.

Riverine populations present several behavioral and social aspects that increase their vulnerability to the acquisition of syphilis. These are situations that turn them weak regarding issues related to morbidity, health access and care, especially those related to sexual and reproductive health. In this scenario, disproportionality in relation to the general population requires a differentiated and combined response among those responsible for fighting syphilis, HIV/AIDS, and other STIs, mainly to ensure universal and equitable access.

Therefore, it is expected that the findings presented contribute to public health actions, given the need to ensure access of vulnerable populations to health services, with the work of nurses in promoting preventive and control strategies, along with early diagnosis and adequate therapeutic adherence. It is also important that they support further research that can broaden the knowledge about riverine populations and their vulnerabilities regarding the acquisition of STIs.

## Financial support

This work was carried out with the support of the Coordination for the Improvement of Higher Education Personnel – Brazil (CAPES) – Financing Code – 001.
